# Social innovation based on collaboration between government and non-governmental organizations in COVID-19 crisis: evidence from Iran

**DOI:** 10.1186/s40249-021-00923-3

**Published:** 2022-01-25

**Authors:** Mehrnaz Moeenian, Abbas Khamseh, Maziyar Ghazavi

**Affiliations:** 1 Department of Technology Management, Science and Research Branch, Islamic Azad University, Tehran, Iran; 2 Department of Industrial Management, Islamic Azad University, Karaj Branch, Karaj, Iran; 3 Department of Industrial Engineering, Islamic Azad University, Qazvin Branch, Qazvin, Iran

**Keywords:** Social innovation, Collaboration with non-governmental organizations, Social policy, COVID-19, Qualitative approach

## Abstract

**Background:**

One of the effective ways to attract social collaboration to provide effective, prompt, and coordinated interventions in emergencies is through social innovation. The present study seeks to identify the factors affecting the implementation of the social innovation plan based on the collaboration between government and non-governmental organizations (NGOs) for saving people’s lives in crises. The initial idea of this research was obtained from the best practice “Every Home Is a Health Base” which was implemented in Iran.

**Methods:**

The Grounded Theory strategy has been used in this study. The statistical population of the study is health experts from the Ministry of Health and Medical Education of Iran. The study time span is during the first half of 2020. Exploratory analysis was used to identify the factors of social innovation. By selecting and reviewing 68 research in-depth, the initial framework was prepared. Then, through a semi-structured interview with experts, the framework was adapted and reviewed. Based on the analysis of the collected data, 39 open codes were extracted and the factors affecting the implementation of the social innovation were identified.

**Results:**

The eight axis codes as the factors affecting the implementation of the social innovation plan based on the collaboration between government and NGOs are as follows: Paying attention to the components of the NGOs collaboration effectiveness, investment to attract NGOs collaboration, the ability to manage the implementation, the ability of networking, the ability of policymaking, providing the necessary cultural and educational infrastructure; Existence of capable legal organizations to solve the executive problems of the plan and facilitate coordination, and controlling, containing and reducing the effects of the crisis, as consequences.

**Conclusions:**

Lessons learned from the COVID-19 pandemic have shown the world that the current governmental and social structures are not efficient enough to respond quickly to the emergence of global challenges. Social innovation is a solution to this problem. The findings of this study also confirm this and identify the factors affecting the implementation of the social innovation plan based on collaboration between governments and NGOs in crises. The results of this research give governments and policymakers an efficient solution by involving NGOs, especially in times of widespread crises. Also, they can be used in planning for social development.

## Background

Innovation is a necessity of civilization. The new circle of world development in the present age is witnessing a wide competition based on innovation to access scarce and limited resources that guarantee the path of long-term and sustainable growth of society. Applying the word innovation to a phenomenon requires that its occurrence cause markedly significant qualitative changes. There are different types of innovations, including technological, economic, business, etc., which are influential in responding to the needs and creating human well-being. However, many needs have not been met. From the point of view of various experts, one of the ways to meet the needs is a kind of dynamic innovation in all areas called social innovation [1–5]. Social innovation is a social goal related to improving the lives of individuals and society to achieve satisfaction through a better, more efficient, effective, fairer, and more sustainable response to existing, new, and especially unmet needs [6]. Social innovation is a new issue which states, that people are always trying to find new solutions to social needs. Hence, part of the appeal of social innovation stems from the fact that it can be used as an “umbrella” to solve social challenges creatively and positively. In other words, social innovations are innovations that are social, both in their means and in their goals, and seek to find new answers to social problems and challenges by identifying and providing new services that improve the lives of people in society [7]. The concept of social innovation consists of two different elements because the social element has various interpretations. Therefore, the relationship between innovation and society is a complex issue [8]. Collaborative partnerships in the field of social innovation are also increasing, with public and private companies and civil society playing a role in these collaborations [9]. This increase in participation in the community, which of course, is to meet the needs of the society, will surge empowerment and expand their scope [10]. Increasing social empowerment also leads to improving social resilience, through which society will become a social system. Social innovation can appear in all organizations and institutions of society and be an achievement for solving social problems creatively. Innovation, especially in times of crisis, requires not only collective intelligence to take effective action toward common goals but also the determination and effort to converge on different ideas as quickly as possible [11]. This convergence can help adopt agile and effective strategies for value creation, even in the current unprecedented pandemic [12].

Awareness of the dangers of a crisis at the right time, provided that prompt and effective action is taken, can be an excellent way to save people’s lives at risk. This awareness has become a significant need for society in times of crisis, and therefore, the role of social innovation to address this need will be very important [10]. The part of social participation by creating convergence in different ideas is essential for the formation of social innovations [11]. Because participation is considered as a social, general, comprehensive, multidimensional, and multicultural process that aims to involve all people to play a role in all stages of development. Therefore, macro-policymakers must look for a way to engage civil society and NGOs in times of crisis. In this regard, the best and most effective way to attract social participation is by using and organizing the neighborhood-based volunteer forces and NGOs. Because while NGOs are part of civil society, their activities and the relationship between them, empower civil society [13]. This neighborhood-based partnership should be designed to win people's trust. Goals and activities should be designed and implemented by NGOs in an acceptable way and in line with the culture and ability of the local people. In recent decades, NGOs have expanded greatly. These organizations are voluntary groups of individuals or organizations that seek to provide public services, support public policy or build capacity for social reconstruction by being independent of governments. The main activities of NGOs are human concerns such as human rights, environmental protection, disaster relief, and development assistance. These organizations provide technical information and expertise that most governments do not have access to [14, 15]. Although most definitions consider NGOs to be non-governmental and active in the private sector, a large part of their activities is among local, national, and international governments. Through communication and affiliation, NGOs meet the goals and aspirations of society and are the best agents for providing leadership for social reconstruction in the developing world [16]. Thus, NGOs seek to influence public policy peacefully in a variety of ways. However, the role of NGOs is not to confront public affairs officials. They are also not an opposition force to the government but act as advisers to serve [17]. In practice, this social participation provides useful information for planners and also helps to identify the cause of the challenges [18]. In this way, it will be a tool to regulate behaviors and, consequently, the possibility of future planning of social processes by the needs.

In the meantime, a scientific review of the current situation may lead to new strategies on how to influence or facilitate government policy change. Given the points and concepts that have been made, the importance of social innovation in a crisis is much more tangible and understandable. In particular a pandemic like COVID-19, which is causing a crisis in the whole world. And, the fact that a pre-arranged tool or formula for controlling such a crisis isn't in hand. The purpose of this study is identifying the factors affecting implementation of the social innovation plan based on the collaboration between government and NGOs in crises especially in the field of health. The importance of the issue stems from the fact that in a pandemic such as the COVID-19, due to the extent, obscurity, and speed of the crisis, the responsible institutions alone cannot succeed in controlling the crisis without the collaboration between government and NGOs. The collaboration between government and NGOs is the process of using the individual or group capabilities of stakeholders to achieve a group goal. In this process, conscious behavior, collective desire, collective acceptance, choice, and shared needs are essential. What important is in a successful collaboration process, is the feeling of need to solve a problem, recognizing that problem, and feeling the need for group cooperation according to the amount of knowledge and ability of individuals and their understanding of existing skills and facilities and their maximum use. A review of previous studies shows that very little attention has been paid to social innovation in academic research [19], and this shortcoming is more pronounced in the issue of social innovation based on collaboration between NGOs and government in a crisis. The novelty of this study to its previous ones [3, 20–32] is to design a social innovation plan based on the collaboration of NGOs and the government in crisis with a wide national or global level. In this study, the collaboration between government and NGOs is discussed in Iran, which is a developing country with a large area and a population of more than eighty million people and a great deal of cultural and ethnic diversity and is under sanctions in terms of international relations. In addition, the crisis refers to the special and unique circumstances of the COVID-19 pandemic. The main purpose of this research is to identify the factors affecting the implementation of the social innovation plan based on the collaboration between government and NGOs in a crisis. Considering that the results of this research can be used in macro policy and government planning in times of crisis and turmoil in the health system, so the research is applied in terms of purpose, also because the grounded theory strategy has been used to extract factors, the research is qualitative in terms of method.

## Methods

**Fig. 1:**
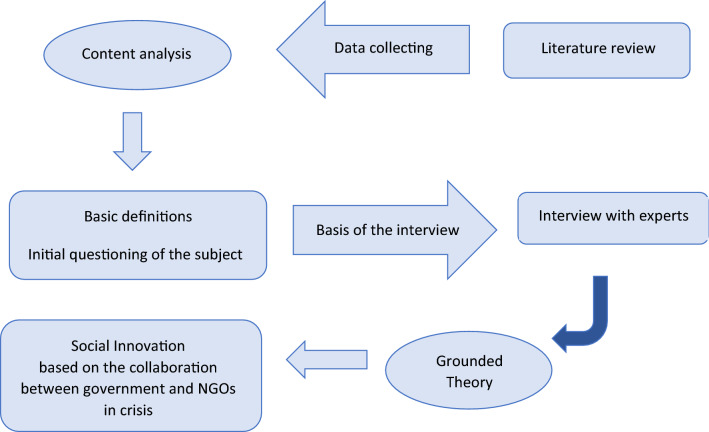
Qualitative research stages using grounded theory. NGO: Non-governmental organizations

The present study is defined to identify the factors affecting the implementation of the social innovation plan based on the collaboration between government and NGOs in a crisis, so in terms of purpose, the research is applied and in terms of method, is qualitative. It also uses the "Grounded Theory" strategy with an inductive approach. The statistical population of the study included health experts working in medical units under the supervision of the Ministry of Health and Medical Education. The mentioned experts are 15 people and have master and higher degrees and more than 10 years of work experience in the field of health. To identify the factors affecting the implementation of the social innovation plan, using the collaboration between government and NGOs, the grounded theory was used, and the qualitative results were analyzed using MAXQDA 2020 software (https://www.maxqda.com). Data collection was done through semi-structured interviews. The researcher tried to analyze and scrutinize the event and phenomenon by using the opinions and knowledge of the most knowledgeable people about the research topic. Figure 1 shows the qualitative stages of the research.

Given that qualitative research does not have specific tests to confirm validity and reliability depend on the stages of the research and the results obtained; therefore, the validity of the method is measured by checking by the interviewees as well as the use of more than two coders [33, 34]. In this regard, the coding was done by the researcher and then reviewed by social innovation experts. Since the selection of experts for the interview was made by snowball method, the findings were re-examined by 10 of the initial interviewees. Finally, the results indicate the existence of appropriate convergence in the opinions of experts. According to Strauss and Corbin (2008), the following criteria (Table 1) are used as questions to assess the validity of a foundation data research that has been used in this research.

**Table 1:** Evaluation of the quality of research findings

Criterion	Description	Action
Fit	Are the findings consistent with the experience of professionals? Can participants see themselves in the story, even if not all the details are relevant to them? Do the findings show them a sense of reality? Do participants and professionals react emotionally to the findings?	Research findings were shared with 10 interviewees. They confirmed it after observing the findings. In addition, the findings were discussed with social innovation experts, which resulted in the assurance of the findings
Applicability	Do the findings provide a new explanation or insight? Can they be used to change performance or add new content to the knowledge base to develop policies?	The findings of this study help policymakers and managers in the field of health and macro-social issues identify the factors affecting the success of social innovation based on the collaboration between government and NGOs in crises and help managers and policymakers in strategic decisions, especially in a crisis
Concepts	Concepts are essential for developing mutual understanding and discussion among professionals; Therefore, the findings are expected to be organized in terms of concepts. As a result, it does not matter how the findings are presented. Rather, it is important that the findings make sense or that they should not be something beyond the mass of interpreted data that makes the reader try to understand it. Of course, concepts must be developed in terms of their characteristics and dimensions to create density and diversity	Attempts were made to make the research findings meaningful and to develop them into meaningful main and sub-categories; Therefore, the research findings were shared with 10 interviewees. After observing the findings, they stated that the findings were understandable to them. In addition, the findings were discussed with social innovation experts, which resulted in the assurance of the findings
Contextualization of concepts	The findings are incomplete without context. Without context, the reader of the research is not able to fully understand the events that took place and why certain meanings are attributed to the events	To this end, an attempt was made to express the research findings in the field that leads to the success of social innovation based on the collaboration between government and NGOs in a crisis
Logic	Is there a logical flow of ideas? Do the findings make sense? Or are there gaps or missing links in logic that confuse the reader? Are the methodological decisions clear enough for the reader to judge whether they are appropriate for data collection and analysis?	To this end, efforts were made to make the research findings have a tangible meaning and the methodological decisions to be completely clear so that the reader can judge whether they are suitable for data collection and analysis
Depth	While the concepts provide a common language for discussion and structure the findings, depth is a descriptive detail that enhances richness and diversity and takes the findings out of the ordinary. In-depth is the concept that distinguishes between insignificant findings and those that have the potential to change policy and practice	To this end, an attempt was made that the concepts in this study provide a common language for discussion and give an organized structure to the data to provide in-depth findings and, as far as possible, more internal layers in social innovation based on popular participation in the statistical community
Variation	Is there diversity in the findings? That is, are there examples of items that do not fit the pattern or have differences in specific dimensions or features?	In this study, an attempt was made to refer to social innovation experts to examine new dimensions of the phenomenon that are different from the dominant pattern that emerged in previous findings
Creativity	Are the findings presented creatively and innovatively? Does this research say anything new? Or put old ideas together in new ways?	For this purpose, in this research, an attempt was made to present the findings creatively and innovatively. This was achieved through a review of multiple data and their frequent analysis
Sensitivity	Has the researcher been sensitive to participants and data? Were the data collection questions obtained through analysis? Or were the concepts and questions created before the data was collected?	In this study, an attempt was made to use a semi-structured interview. So, several questions were asked from the beginning, but during the interview and data collection process, new questions were formed and guided the research
Evidence of memos	Because the researcher cannot recall all the insights, questions, and depth of thought that go through the analysis, notes are one of the most essential steps. Notes should grow in depth and degree of abstraction as research progresses	In this research, an attempt was made to write down points that come to the researcher's mind during the interview process. Also, during the analysis process, important points were entered in the notes section of MAXQDA software

NGO: Non-governmental organizations

### Designing the basis of the interview

In the first stage, to collect data, 68 references, including articles and books related to social innovation, were examined. For this purpose, to access the documents, researches entitled "Social Innovation" were first extracted from valid databases. They were then screened several times. Finally, the authors reached a theoretical consensus, and the final documents were selected. The indicators extracted from these documents are given in Table 2.

**Table 2:** Summary of variables extracted from literature review and research

Variable									
Source	Identifying a problem or need	Outbreak idea, method, or answer	Interest, decision, or encouragement to get involved	Shaping collaboration and partnership	Stimulating the process through a social goal	Testing of samples and choosing the right idea	Providing infrastructure	Identifying risk factors	Running on a large scale
Moscibrodzki et al. [35]	✓								
Kpokiri et al. [36]				✓			✓		
Rollin and Vincent [27]		✓				✓	✓		✓
Mulgan et al. [22]	✓	✓				✓			✓
Murray et al. [20]	✓	✓				✓			✓
Young Social Innovators (2010)			✓	✓					
Hubert [26]	✓	✓							✓
Assogba [28]		✓	✓		✓				✓
The Young Foundation [23]		✓				✓			✓
Pitt Catsouphes et al. [30]		✓	✓			✓	✓		
Herrera and Alarilla [25]						✓			
Norman et al. [21]		✓	✓		✓	✓			✓
Hahn and Andor [29]		✓				✓			✓
Raffeld et al. [3]		✓		✓					
Dako-Gyeke et al. [31]		✓	✓	✓					
Castro-Arroyave and Duque-Paz [32]				✓			✓	✓	

Variable									
Source	Deployment and implementation	Institutionalization and Sustainability	Systematic changes	Intersectoral and interdisciplinary exchange	Learning and empowerment	Measuring effectiveness	Culture building	Community mobilization	Digital Social Innovation
Moscibrodzki et al. [35]				✓				✓	✓
Kpokiri et al. [36]				✓	✓				
Rollin and Vincent [27]	✓	✓							
Mulgan et al. [22]					✓				
Murray et al. [20]			✓			✓			
Young Social Innovators (2010)			✓	✓					
Hubert [26]	✓					✓			
Assogba [28]	✓	✓							
The Young Foundation [23]	✓								
Pitt Catsouphes et al. [30]	✓	✓				✓			
Herrera and Alarilla [25]	✓	✓	✓		✓				
Norman et al. [21]	✓		✓			✓			
Hahn and Andor [29]	✓			✓					
Raffeld et al. [3]		✓			✓				
Dako-Gyeke et al. [31]	✓	✓			✓	✓			
Castro-Arroyave and Duque-Paz [32]				✓	✓		✓		

### Implementing grounded theory strategy

In the second stage, to identify the dimensions of the social innovation based on the collaboration between government and NGOs in the crisis of COVID-19, the grounded theory and semi-structured interviews with 15 health experts, which were determined by the snowball method, were used. These experts had master's and higher education and had an average of 17 years of experience working in medical units under the supervision of the Ministry of Health and Medical Education, and had responsibility in the national program "Every Home Is a Health Base" to control and manage, the COVID-19 pandemic, in a neighborhood and family-centered manner. From the tenth person onwards, the data analysis did not lead to the discovery of new concepts and categories. However, to ensure theoretical saturation, five more interviews were conducted, and the data related to them were analyzed. The coding steps in grounded theory include three steps of open, axial, and selective coding [33]. Each of these steps is described below.

Step 1: Open Coding: This step of the grounded theory method is performed immediately after the first interview; In other words, after each interview, the researcher begins to find concepts and select appropriate labels for them and combine related concepts. According to Strauss and Corbin (1998), the steps of open coding are:
  1. Analysis and coding: At this stage, the researcher must pay attention to coding and all events. Many codes may be extracted from an interview or text, but when the data is reviewed, new coding counts and final codes are identified.
  2. Discover the categories: At this stage, the concepts themselves are classified based on similar topics, which is called categorization (theme building). The titles we assign to categories are more abstract than the concepts that make up that category. Classes have high conceptual power because they can gather concepts around their axis. Introductory titles were chosen mainly by the researcher himself and tried to have the most relevance and consistency with the data it represents. Another important source is the terms used by research participants and can be used by the researcher.
  3. Description of categories according to their characteristics: To make the categories clearer, their properties are stated in the next step.
  4. Open coding table: Includes a table of primary codes extracted from the interviews and a table of categories extracted from the concepts along with their secondary codes.
Step 2: Axial coding: In the second stage of coding, which is called axial coding, the researcher selects one of the categories as the axial category and explores it as the axial phenomenon in the center of the process and determines the relationship of the other categories with it.

## Introducing the best practice "Every Home Is a Health Base"

Paying attention to the best practice in policymaking and related research is an essential point that can give more credibility to the results [37]. Therefore, in this research, the national program "Every Home is a Health Base" as a best practice [38], in the field of information and pandemic control was reviewed and modeled. In this project, the management and control of the COVID-19 pandemic were designed and implemented jointly by the Ministry of Health & Medical Education and the NGOs in a neighborhood and family-centered manner. In the first half of 2020, a senior official from the Ministry of Health & Medical Education launched the idea of the "Every Home is a Health Base" program with the primary goal of attracting public and NGO participation in efforts to control the pandemic. In this project, the method of attracting public participation has been through the selection of a health ambassador for each household, and a neighborhood health interface for every 40 household ambassadors, (ambassadors were from NGOs) focusing on mobilizing neighborhood facilities, on improving social (including health), the economic and cultural status of the community. In this regard, using all the capacities of the country, especially the NGOs, to help the Ministry of Health, which has been in control of the pandemic, by the implementation of the plan to hand over controlling COVID-19, to the people of the neighborhoods by using the coordination and management of active units in the neighborhoods (health base in urban areas / rural health house and mobilizing neighborhoods) and extensive participation of other stakeholders such as Red Crescent and NGOs, was used as a suitable strategy to control the disease at the neighborhood level. This project is a clear example of attracting public participation, cross-sectoral coordination, planning based on the needs of neighborhoods (Bottom-up Planning), and optimal use of the potential of the country's health care network system. A very important task of planning and coordination is to educate the public to improve the knowledge and skills of the people. On the complexity of the Covid 19 pandemic challenge, Morawska‑J (2021) [39] expresses that social innovation is an element of a regional innovation system that universities can use purposefully to implement. Also, van Niekerk et al. (2021) [40] showed in their research that universities play a key role in the success of social innovation for health, especially in low- and middle-income countries. In the mentioned best practice, the University of Medical Sciences has played a significant role in training the people's forces and NGOs and the success of the project. To face COVID-19, relying on the program "Every home is a health base", and taking advantage of all local facilities and extensive participation of mobilization NGOs and people, was entrusted to the Central Committee under the responsibility of the University of Medical Sciences. This was done through support, care, and monitoring teams [41].

## Results

In this research, social innovation based on the collaboration between government and NGOs in crises is the main category, and the effectiveness of NGOs collaboration in the implementation of the social innovation plan is the axial code that was extracted from open codes based on the inductive approach, is presented in Table 3. The following five headings explain the connection between other categories and the axial category [34].

**Table 3:** Coding based on Strauss and Corbin approach to the axial phenomenon (axial category)

Macro category	Axis code	Open codes	Sample interview code
Social innovation based on the collaboration between government and NGOs in a crisis	The effectiveness of NGOs collaboration in the implementation of the social innovation plan	The executive capacity of NGOs, motivation, and commitment of NGOs, education, and experience of NGOs’ members, ability to communicate effectively by NGOs, number of NGOs, having a spirit of sacrifice and self-dedication of NGOs’ members, developing and promoting a culture of participation in society	P1, P2, P3, P4, P5, P6, P7, P8, P9, P10, P11, P12, P13, P14, P15

NGO: Non-governmental organizations

1. Axial phenomenon: which is also called the main category, is a phenomenon that is the main axis of research (Table 3).
2. Causal conditions: These conditions cause the formation of axial phenomenon or category. These conditions are a set of categories and their characteristics that affect the axial category. In this research, the micro category based on open codes of interviews and documents (in the form of Table 4) is including investment, to attract NGOs’ collaboration in the implementation of the social innovation plan.

**Table 4:** Coding based on the approach of Strauss and Corbin on causal conditions

Macro category	Axis code	Open codes	Sample interview code
Social innovation based on the collaboration between government and NGOs in a crisis	investment, to attract NGOs collaboration in the implementation of the social innovation plan	Government fiscal policies in support of NGOs collaboration, the amount of investment in the social innovation plan, the continuation of investment in the social innovation plan during the crisis, financial support in the form of incentive packages, educational support based on experts in the field, support for research projects on social innovation in the crisis	P2, P3, P4, P6, P8, P9, P10, P11, P12, P13, P14, P15

NGO: Non-governmental organizations
3. Strategies: 1. Express purposeful behaviors, realities, and interactions that are achieved under the influence of intervening conditions and the prevailing context. In this study, the ability to manage the implementation of the social innovation plan and the ability to network are two micro categories that were identified by decoding. The strategies are presented in Table 5.

**Table 5:** Coding based on Strauss and Corbin approach to strategies

Macro category	Axis code	Open codes	Sample interview code
Social innovation based on the collaboration between government and NGOs in a crisis	Ability to manage the implementation of the social innovation plan	Ability to plan project implementation, ability to budget and allocate project funding, ability to evaluate project effectiveness, ability to control and monitor project implementation, identify corrective actions, ability to act quickly and promptly when risks and opportunities arise	P1, P2, P3, P4, P5, P6, P7, P9, P10, P11, P12, P13, P14
	Ability to network	Attract involvement of organizations effective in project implementation, knowledge transfer and sharing, knowledge networking, support management, and project implementation	P1, P2, P4, P5, P6, P8, P9, P10, P11, P12, P13, P15

NGO: Non-governmental organizations
4. Ruling context or contextual conditions: The specific conditions that affect strategies are called contexts, and it is difficult to distinguish them from causal conditions. These conditions include a set of concepts, categories, or contextual variables; in contrast, causal conditions are a set of active variables. Sometimes highly related variables are classified under causal conditions and less relevant variables are classified under the prevailing context. In this research, contextual factors include two micro categories of ability to policymaking overall social innovation plan and providing the necessary cultural and educational infrastructure (Table 6).

**Table 6:** Coding based on the approach of Strauss and Corbin on contextual conditions

Macro category	Axis code	Open codes	Sample interview code
Social innovation based on the collaboration between government and NGOs in a crisis	Ability to policymaking overall social innovation plan	Ability to identify crisis features and characteristics, Ability to identify possible solutions to crisis management, Ability to select optimal solutions, Ability to organize for implementation, identify risks and opportunities for project implementation, Ability to define project performance evaluation indicators	P1, P2, P3, P4, P5, P6, P7, P8, P9, P10, P11, P12, P13, P14, P15
	Providing the necessary cultural and educational infrastructure	Public awareness (media infrastructure including mass media and cyberspace, educational packages, local advertising), development of educational topics in the curriculum of primary and secondary schools, using the public acceptance of influential people in different strata and regions to spread the culture of participation in the community	P1, P2, P3, P4, P5, P7, P8, P9, P10, P11, P12, P13, P14, P15

NGO: Non-governmental organizations
5. Intervening conditions: There are situations in which strategies are affected. These conditions constitute a set of mediating variables. Intervening conditions are structural conditions that facilitate or limit the intervention of other factors and have a causal and general nature. Based on the open codes, the micro category in this step includes the existence of capable legal organizations to solve the executive problems of the plan and facilitate coordination. The coding for the intervention conditions is given in Table 7.

**Table 7:** Coding based on Strauss and Corbin's approach to intervening conditions

Macro category	Axis code	Open codes	Sample interview code
Social innovation based on the collaboration between government and NGOs in a crisis	Existence of capable legal organizations to solve the executive problems of the plan and facilitate coordination	Facilitating the provision of necessary budget and financial resources, involving other organizations and institutions to solve unforeseen problems of the project, facilitating access to support resources	P2, P3, P4, P6, P7, P8, P9, P10, P11, P12, P13, P14

NGO: Non-governmental organizations
6. Consequences: Some categories represent the results and consequences that result from the adoption of strategies. This coding method, which is called the "paradigm model" of axial coding, has been proposed by Strauss and Corbin and is called axial because the coding is done around the "axis" of a category. The micro category in this section obtained from open coding includes controlling, containing, and reducing the effects of the crisis. The coding of the consequences is given in Table 8.

**Table 8:** Coding based on Strauss and Corbin's approach to consequences

Macro category	Axis code	Open codes	Sample interview code
Social innovation based on the collaboration between government and NGOs in a crisis	Controlling, containing, and reducing the effects of the crisis	Measuring the level of acceptance and public cooperation with the implementers of the project during the implementation, measuring the statistics of infected patients during the implementation, reducing the death rate by informing the necessary measures in case of infection	P1, P2, P3, P4, P5, P6, P7, P8, P9, P10, P11, P12, P13, P14, P15

NGO: Non-governmental organizations

**Fig. 2:**
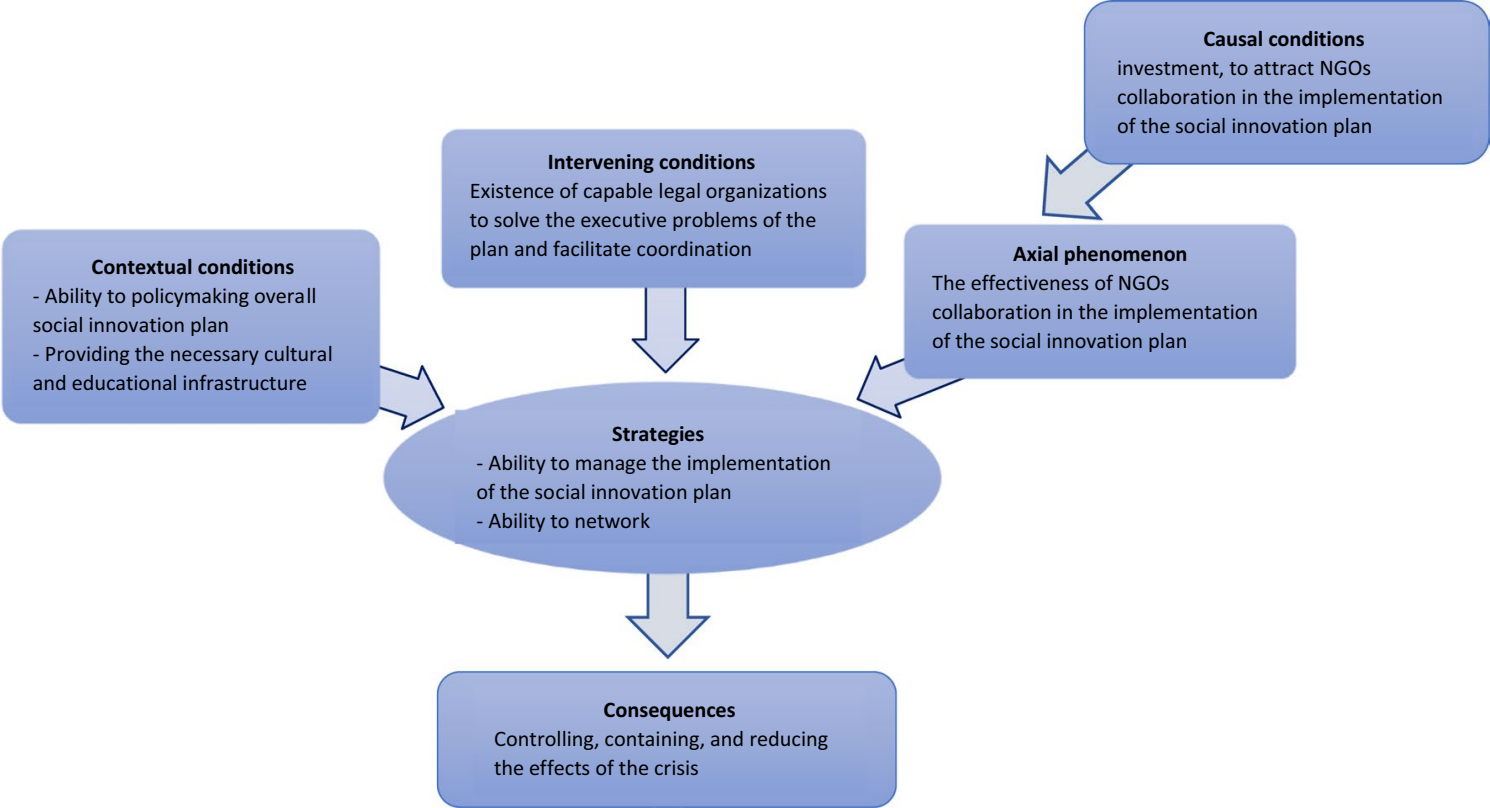
Axial coding designed paradigm model. NGO: Non-governmental organizations

Finally, at this stage of the qualitative data results, the axial coding paradigm is developed. Figure 2 shows the extracted axial coding paradigm.

**Fig. 3:**
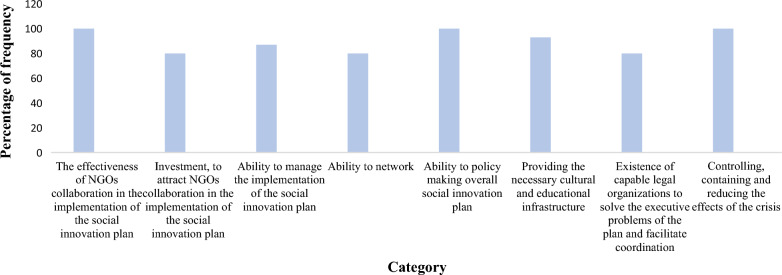
Frequency of social innovation categories based on the collaboration between government and NGOs in a crisis in MAXQDA software

At the next stage of coding, the grounded theorist writes a theory of the relationships between the categories in the axial coding model. In this research, for the dimension of integration and improvement of categories, we achieved one selected code of the effectiveness of NGOs’ collaboration in the implementation of the social innovation plan. Table 9 shows the identified codes and Fig. 3 shows the frequency of categories.

**Table 9:** Identified codes related to social innovation based on the collaboration between government and NGOs in a crisis

Selective code	Axis Code	Open Codes
Social innovation based on the collaboration between government and NGOs in a crisis	The effectiveness of NGOs collaboration in the implementation of the social innovation plan **(C1)**	The executive capacity of NGOs **(C11)**, motivation and commitment of NGOs **(C12)**, education and experience of NGOs’ members **(C13)**, ability to communicate effectively by NGOs **(C14)**, number of NGOs **(C15)**, having a spirit of sacrifice and self-dedication of NGOs’ members **(C16)**, developing and promoting a culture of participation in society **(C17)**
	investment, to attract NGOs collaboration in the implementation of the social innovation plan **(C2)**	Government fiscal policies in support of NGOs collaboration **(C21)**, the amount of investment in the social innovation plan **(C22)**, the continuation of investment in the social innovation plan during the crisis **(C23)**, financial support in the form of incentive packages **(C24)**, educational support based on experts in the field **(C25)**, support for research projects on social innovation in the crisis **(C26)**
	Ability to manage the implementation of the social innovation plan **(C3)**	Ability to plan project implementation **(C31)**, ability to budget and allocate project funding, ability to evaluate project effectiveness **(C32)**, ability to control and monitor project implementation **(C33)**, identify corrective actions **(C34)**, ability to act quickly and promptly when risks and opportunities arise **(C35)**
	Ability to network **(C4)**	Attract involvement of organizations effective in project implementation **(C41)**, knowledge transfer and sharing **(C42)**, knowledge networking **(C43)**, support management, and project implementation **(C44)**
	Ability to policymaking overall social innovation plan **(C5)**	Ability to identify crisis features and characteristics **(C51)**, Ability to identify possible solutions to crisis management **(C52)**, Ability to select optimal solutions **(C53)**, Ability to organize for implementation **(C54)**, identify risks and opportunities for project implementation **(C55)**, Ability to define project performance evaluation indicators **(C56)**
	Providing the necessary cultural and educational infrastructure **(C6)**	Public awareness (media infrastructure including mass media and cyberspace **(C61)**, educational packages **(C62)**, local advertising **(C63)**, development of educational topics in the curriculum of primary and secondary schools **(C64)**, using the public acceptance of influential people in different strata and regions to spread the culture of participation in the community **(C65)**
	Existence of capable legal organizations to solve the executive problems of the plan and facilitate coordination **(C7)**	Facilitating the provision of necessary budget and financial resources**(C71)**, involving other organizations and institutions to solve unforeseen problems of the project **(C72)**, facilitating access to support resources **(C73)**
	Controlling, containing, and reducing the effects of the crisis **(C8)**	Measuring the level of acceptance and public cooperation with the implementers of the project during the implementation **(C81)**, measuring the statistics of infected patients during the implementation **(C82)**, reducing the death rate by informing the necessary measures in case of infection **(C83)**

## Discussion

Lessons learned from the widespread COVID-19 crisis have shown the world that the current economic and social structures are not efficient enough to respond quickly and effectively [35] to such conditions and need to be fundamentally revised and transformed. To this end, the post-Covid world should use a redistributive framework and surveillance systems as the basis for development rather than development by focusing on economic growth [42]. In this regard, one of the categories that has grown after the emergence of this global challenge is social innovation, and the necessity and importance of having models of social innovation that are operational and effective in times of crisis were increasingly raised as an efficient solution. The term innovation has become a key concept among policymakers around the world due to its widespread use, adaptability, and direct connection to new, creative, and positive ideas [43–46]. Previous research shows that the concept of innovation through "learning" is attractive to policymakers for a better understanding of uncertain future challenges and the possibility of "easy" planning [46]. In social innovation, policy projects are used as a tool to align policy issues with local and community needs [47].

In the present study, to identify the factors affecting the implementation of the social innovation plan based on collaboration between government and non-governmental organizations in a crisis, the theoretical foundations were studied, and field studies were conducted by identifying relevant categories and codes, not only to assist policymakers and managers in macro-strategic decisions but also to create a plan that can be used for possible future crises. The results of this study proved that social innovation is a concept with a much broader application of local partnerships to meet the needs in small areas and, this concept can be used effectively at the national level with appropriate speed and agility. It is worth mentioning that the plan has several applications, including the development of evidence for a cognitive and multidimensional approach to community empowerment and, consequently, the formation of social innovations. In this research, in the first step, semi-structured interviews were conducted based on the six sections of the grounded theory. Then, based on the analysis of qualitative findings in three stages of open, axial, and selective coding, six categories of causal (Investment, to attract NGOs collaboration in the implementation of the social innovation plan), axial (The effectiveness of NGOs collaboration in the implementation of the social innovation plan), strategies (Ability to manage the implementation of the social innovation plan and Ability to network), intervening (Existence of capable legal organizations to solve the executive problems of the plan and facilitate coordination), contextual (Ability to overall policymaking social innovation plan and Providing the necessary cultural and educational infrastructure) and consequences (Controlling, containing and reducing the effects of the crisis) were identified. The results of the present study are in line with the findings of the research of Moscibrodzki et al. (2021), Kpokiri et al. (2021), Morawska‑J (2021), Rollin and Vincent (2007), Mulgan et al. (2007), Murray et al. (2010), Young Social Innovators (2010), Hubert (2010), Assogba (2010), The Young Foundation (2012), Pitt Catsouphes et al. (2012), Herrera and Alarilla (2013), Norman et al. (2013), Hahn and Andor (2013), Raffeld et al. (2015), Dako-Gyeke et al. (2020) and Castro-Arroyave and Duque-Paz (2020).

In this research, the factors were identified with a qualitative approach. In future research, quantitative methods can be used by other researchers. Social innovations in a crisis can also be explored for NGOs' cooperation at the transnational level with international institutions. Also, specifically the role of universities, can be discussed and confronted with the importance of NGOs as another important sector in social innovation in health at the time of crises.

## Conclusions

The research findings indicate that the effectiveness of NGOs collaboration in the implementing of the social innovation plan, Ability to policymaking overall social innovation plan, and Control, contain and reduce the effects of the crisis are the most critical factors in social innovation based on the collaboration between government and NGOs. The results of this study provide the basis for the development of social innovations based on the collaboration between government and NGOs that leads to sustainable development in communities.

Based on the research results, it is suggested:

1. Support laws should be enacted by the legislative authorities to strengthen and facilitate the performance of NGOs.
2. Institutions should be established by the government to facilitate communication with NGOs.
3. In the context of other crises with a more limited scope (such as earthquakes and floods, etc.), should be focused on how to strengthen and organize and integrate NGOs and government centers.
4. For the participation process to be sustainable, other stakeholders and investors, such as private companies and academic associations, should be identified, and their opinion can be attracted for participation.
5. To increase the effectiveness of social participation, it is necessary to align the policies of other executive bodies with the goals of NGOs.

## Data Availability

The datasets used and analyzed during the current study are available from the corresponding author on reasonable request.

## Abbreviation

NGO: Non-governmental organization
